# Clinical Characterization of Oculomotricity in Children with and without Specific Learning Disorders

**DOI:** 10.3390/brainsci10110836

**Published:** 2020-11-11

**Authors:** Carmen Bilbao, David P. Piñero

**Affiliations:** 1 Department of Optometry, Policlínica Alto Aragón, 22003 Huesca, Spain; carmenbill0@gmail.com; 2 Group of Optics and Visual Perception, Department of Optics, Pharmacology and Anatomy, University of Alicante, 03690 San Vicente del Raspeig-Alicante, Spain; 3 Department of Ophthalmology, Vithas Medimar International Hospital, 03016 Alicante, Spain

**Keywords:** specific learning disorders, DEM, NSUCO, oculomotor anomalies, saccades, dyslexia

## Abstract

Children with specific learning disorders have been associated with oculomotor problems, with their analysis even suggested to be a potential diagnostic tool. A prospective non-randomized comparative study evaluating 59 children (6–13 years old) divided into three groups was conducted: a control group (CG) including 15 healthy emmetropic children; a group of 18 healthy children with oculomotor abnormalities (OAG); and a group of 26 children diagnosed with specific learning disorders (LDG). In all groups, besides a complete eye exam, oculomotricity was characterized with two clinical tests: Northeastern State University College of Optometry’s Oculomotor (NSUCO) and Developmental Eye Movement (DEM) tests. Concerning the NSUCO test, lower ability, precision, and head/body movement associated scorings were obtained for both smooth pursuits and saccades in OAG and LDG when compared to the CG (*p* < 0.001). Likewise, significantly longer time needed to read the horizontal sheet of the DEM test and a higher DEM ratio were found in OAG and LDG compared to CG (*p* ≤ 0.003). No differences between LDG and OAG were found in the performance with the two oculomotor tests (*p* ≥ 0.141). Oculomotor anomalies can be present in children with and without specific learning disorders, and therefore cannot be used as diagnostic criteria of these type of disorders.

## 1. Introduction

Oculomotricity has been studied in detail since the beginning of the 20th century [1], with much knowledge on this issue acquired by researchers in recent years [2]. However, the connection between experimental findings and their clinical application is still limited, which makes the implementation of an evidence-based clinical practice in oculomotor dysfunctions difficult [3]. For this reason, more controlled clinical studies are needed to define normality patterns for those tests used to evaluate oculomotricity in clinical setting and to develop accurate diagnostic criteria for oculomotor dysfunctions.

Two main types of eye movements are commonly used to characterize oculomotricity in clinical practice: saccades and pursuits. Saccadic movements are rapid, ballistic movements of the eyes that abruptly change the point of fixation [4]. The saccade is the fastest type of movement that the human body can generate [5], with an average speed of 100 to 800° per second, and frequency of 100,000 of saccades per day [4,6]. They are crucial in the development of reading [7]. Altered saccades can be found in some patients due to aging [8], alcohol consumption [9], intake of some medications [10], neurological alterations [6,11] or lack of natural sleep [12]. Smooth pursuits, are smooth coordinated tracking movements of the eyes designed to keep a moving stimulus on the fovea [4]. Their main objective is to conduct a fluid search to maintain the same speed of the object, thus minimizing distortion of the retinal image [13]. If the object changes direction unexpectedly, the eye loses the object for 200 ms, causing a refixation [14]. These types of movements can be also distorted by some neurological alterations [15].

One group of patients that have been normally associated to oculomotor abnormalities are children with learning disorders [16]. Indeed, several studies have reported some specific differences in oculomotor patterns in these children compared to others with no learning disorders, even concluding that these differences may be a potential ocular biomarker of specific learning disorders [17,18,19,20]. However, the diagnosis of oculomotor analysis in this type of patients is still controversial, as no clear range of normality for the parameters used to characterize ocular motility has been clearly defined, and no gold standard or reference test has been established [16]. Furthermore, the real impact of this potential oculomotor anomaly on daily activities has not been clearly determined. It should be considered that children with specific learning disorders manifest significant difficulties in the acquisition and use of listening, speaking, reading, writing, reasoning or mathematics skills independent of whether a visual deficit is present or not [21]. Likewise, reading difficulties related to oculomotor alterations have been reported in children without specific learning disorders [22]. The aim of the current study was to compare the oculomotor patterns characterized by widely available clinical tests (not requiring the acquisition of technology) in healthy children without visual problems, healthy children with oculomotor alterations, and children with a diagnosis of learning disorders in order to determine which type of differences can be found and how this information can be used to make clinical decisions.

## 2. Materials and Methods

### 2.1. Patients

This was a prospective non-randomized comparative study evaluating a total of 59 children with ages ranging from 6 to 13 years old at the Department of Optometry of the Policlínica Alto Aragón (Huesca, Spain). The research adhered to the principles of the Declaration of Helsinki and was approved by the ethics committee of the University of Alicante (exp. UA-2018-02). Written informed consent was obtained from the parents of the children after they were given an explanation about the study protocol, risks, and benefits. Three groups were differentiated according to the following characteristics:

- Control group (CG): included 15 healthy children recruited from various schools in the province of Huesca. The inclusion criteria for this group were emmetropic children achieving an uncorrected distance visual acuity (UDVA) of 0.00 logMAR (20/20 Snellen).
- Group of healthy children with oculomotor abnormalities (OAG): included 18 children that attended the Optometry Unit of Policlínica Alto Aragón Polyclinic (Huesca, Spain). Inclusion criteria for this group were children aged from 6 to 14 years old, wearing their refractive correction if needed for more than 6 months, absence of strabismus or non-strabismic binocular or accommodative disorders, and presence of an oculomotor anomaly. This anomaly was detected using the DEM (Developmental Eye Movement) test and its normative data [23].
- Group of children diagnosed with a specific learning disorder (LDG): 15 of them showed dyslexia, 6 showed a development coordination disorder (DCD) or dyspraxia, and 5 of them showed an attention deficit/hyperactivity disorder (ADHD). A speech therapist, a psychologist and a pediatrician evaluated the conditions of these children and performed a diagnosis according to the DSM-5 diagnostic criteria [24]. According to previous studies, these children are expected to show oculomotor abnormalities in most of cases [17,18,19,20,25,26,27,28,29].

The following exclusion criteria were established for the three groups evaluated: aged over 14 years old, active ocular disease, previous ocular surgeries, diagnosis of a neurological or neurodegenerative disorder, history of neurophthalmic disease, previous vision therapy, and inclusion in any other clinical study.

### 2.2. Visual Examination

A complete visual examination was performed after an exhaustive ophthalmologic evaluation of ocular health that included: measurement of uncorrected and corrected distance visual acuity (UDVA and CDVA, respectively); manifest and cycloplegic refraction; measurement of near point of convergence (NPC) using the Lang bar; Worth four dot test; cover test at far (5 m) and near (40 cm) distances; measurement of stereopsis (Wirt test); and evaluation of oculomotricity using two different clinical tests: NSUCO (Northeastern State University College of Optometry’s Oculomotor) and DEM (Developmental Eye Movement) tests. These two tests have demonstrated their reliability and precision in the assessment of oculomotricity in children [30,31]. It should be considered that no videoculographic or eye-tracking registry was available in our clinical setting. All measurements were performed by the same experienced examiner (CB).

### 2.3. Clinical Analysis of Oculomotricity

The NSUCO test is a standardized procedure with scoring criteria [32]. The test subjectively evaluates eye and saccadic movements considering four performance areas that are classified into chasing and saccade skills:

- Ability. Can the individual take the assigned test?
- Accuracy. What is the quality of execution?
- The level of head movement the patient uses to perform the task. Is head movement spontaneous when doing the task?
- The level of body movement used.

Therefore, a total of eight areas of performance are evaluated, four areas for smooth pursuits and four areas for saccades. The protocol consists of rating each area of analysis from one to five, with five being the most optimal performance. It should be considered that attention is a factor that is clearly involved in the skills evaluated by the test, with the requirement of maintaining the attention focus as much as possible during the entire sequence.

The test was conducted at 40 cm binocularly, with the patient sitting in front of the examiner. As fixation stimuli, small colored spheres of 0.5 cm in diameter and mounted on a rod were used. Smooth pursuits were evaluated by moving the stimulus circumferentially (20 cm of diameter approximately) clockwise and counterclockwise, whereas saccades were evaluated by asking the patient to alternate fixation between two stimuli separated horizontally 20 cm [31,32]. According to the examiner’s observation, the scoring was determined according to the following criteria [31,32]:

- Smooth pursuits:
  ○ Patient’s ability of performing two rotations (ability): cannot complete half rotation (1 point); half rotation completed (2 points); rotation completed in each direction (3 points); two rotations completed in one direction (4 points); and two rotations completed (5 points).
  ○ Patient’s ability of performing two rotations without refixations (accuracy): more than 10 refixations (1 point); 5 to 10 (2 points); 3–4 (3 points); 2 refixations or fewer (4 points); and no refixations (5 points).
  ○ Patient’s ability of performing two rotations without head or body movements: exaggerated body or head movement (1 point); large or moderate movement (2 points); slight movements but constant (3 points); slight movements but intermittent (4 points); and no head or body movements (5 points).
- Saccades:
  ○ Patient’s ability of performing 5 cycles of change of fixation between the two stimuli presented (ability): 1 cycle or no ability (1 point); 2 cycles (2 points); 3 cycles (3 points); 4 cycles (4 points); and 5 cycles (5 points).
  ○ Patient’s ability of performing 5 cycles of change of fixation without correcting refixations (accuracy): very significant hyper- or hypometric movements (1 point); large to moderate hyper- or hypometric movements (2 points); slight hyper or hypometric movements but constant (3 points); slight hyper or hypometric movements but intermittent (4 points); and no correcting refixations (5 points).
  ○ Patient’s ability of performing 5 cycles of change of fixation without head or body movements: same scoring as for smooth pursuits.

The DEM examination is a validated test to evaluate oculomotricity during reading in children from 6 to 14 years old [30], although an adult version has been developed [33]. Specifically, the test consists of 4 sheets that the patient must read: a preliminary sheet that is used to observe the ability to read numbers (if the patient cannot read the demo sheet, the test could not be performed), two sheets with two columns of numbers listed vertically which the patient must read without placing their index finger as an indicator (“A” and “B” sheets), and a sheet with letters displayed in horizontal lines (“C” sheet). The examiner recorded the time needed by the patient to read the A, B and C sheets, as well as the number and type of mistakes. With these data, a ratio (Horizontal Time/Vertical Time) was calculated, and the result was compared to the normative data [30]. Four oculomotor patterns can be detected with the DEM system:

- Type 1: No oculomotor dysfunction. This occurs when the vertical and horizontal test times are within the normal range.
- Type 2: Oculomotor dysfunction. The vertical time is within the normal range, but the horizontal time is below the normal range according to the patient’s age.
- Type 3: Automaticity dysfunction. Vertical and horizontal times are long but of similar magnitude, giving a ratio below the normal range.
- Type 4: Automaticity dysfunction and oculomotor dysfunction. A combination of a long vertical time and an extremely long horizontal time [30].

### 2.4. Statistical Analysis

The statistical data analysis was performed using the software SPSS version 15.0 for Windows (SPSS, Chicago, IL, USA). The Kolmogorov–Smirnov test confirmed that most of samples did not follow a normal distribution and therefore non-parametric tests were used. The Kruskal–Wallis test was used to analyze the significance of differences in a great variety of clinical variables between the groups involved in the study, with a post-hoc analysis performed using the Mann–Whitney test adjusted with the Bonferroni correction. All statistical tests were 2-tailed, and *p*-values less than 0.05 were considered to be statistically significant.

## 3. Results

The sample was comprised of 59 children (28 girls and 31 boys) with ages ranging from 6 to 11 years old. As previously mentioned, three groups were differentiated, CG (15 children), OAG (18 children), and LDG (26 children), with no significant differences in gender distribution (*p* = 0.334). The main characteristics of these three groups are summarized in Table 1. As shown, statistically significant differences between groups were only found in stereopsis (*p* < 0.001) and the measurement of the phoria at near distance (*p* = 0.047). Specifically, lower levels of stereopsis were found in the LDG compared to the other two (*p* ≤ 0.018). Likewise, there was a trend to exophoria in the LDG compared to the CG (*p* = 0.043).

Table 2 summarizes the results of oculomotor examination with the NSUCO test in the three groups of children evaluated. As shown, statistically significant differences were found between groups in the scoring of the three categories evaluated (ability, precision and head/body movement associated) for smooth pursuits and saccades (*p* < 0.001). Specifically, lower ability, precision and movement head/body associated scorings were obtained for both smooth pursuits and saccades in the OAG when compared to the CG (*p* < 0.001). Likewise, lower NSUCO scorings were obtained in the LDG when compared to the CG (*p* < 0.001). However, no statistically significant differences were found between the OAG and LDG in ability, precision and head/body movement associated scorings for both smooth pursuits and saccades (*p* ≥ 0.141).

Table 3 summarizes the results of oculomotor examination with the DEM test in the three groups of children evaluated. Statistically significant differences were found between groups in time sheet C, DEM ratio, type of DEM pattern, and percentile corresponding to horizontal time (*p* < 0.001). Specifically, significantly longer time sheet C and higher DEM pattern values and ratios, as well as lower percentiles for horizontal time were found in the OAG and LDG compared to the CG (*p* ≤ 0.003). Likewise, significantly longer time sheet B values were found in the LDG compared to the CG (*p* = 0.049).

Concerning the relationship between the measurement of the phoria at near distance with the cover test and the oculomotor parameters evaluated with the NSUCO and DEM tests, they were not significantly correlated in the OAG group (−0.165 ≤ *r* ≤ 0.371, *p* ≥ 0.130). In the LDG group, a poor, although statistically significant, correlation was found between the magnitude of phoria at near distance and the NSUCO score related to the movement of the body and head while performing saccadic movements (*r* = 0.475, *p* = 0.014).

## 4. Discussion

Eye movements and binocular function have been studied for many years in groups of children with learning disorders, reporting oculomotor abnormalities in this population [17,18,19,20,34]. Considering the relevance of saccades for reading activities [7], it has been hypothesized about the potential contribution of oculomotor abnormalities in specific learning disorders to reading difficulties, especially in dyslexia [16]. Likewise, visual exercises to promote oculomotor functionality have been suggested to be useful for improving reading performance in children with specific learning disorders [35,36]. Some authors have even suggested that the reaction time of the saccadic stimulus may be a possible biomarker for the diagnosis of ADHD [27]. However, oculomotor deficits are not only restricted to children with specific learning disorders; they are compatible with some functional alterations [37] or even some neurological diseases [11]. Furthermore, some results have been provided supporting the hypothesis that abnormal eye movements are more likely to be an effect and not the cause of reading difficulties [38]. This study aimed at demonstrating that oculomotor anomalies defined according to two clinical tests not requiring advanced technology can be present in healthy children with and without reading difficulties in the same magnitude and extent as they are present in children with specific learning disorders.

Although videoculography has been used in several studies as an objective mode of characterizing oculomotor anomalies, no clear and validated diagnostic criteria have been defined for the different oculomotor aspects explored with this advanced technology [16]. For this reason, the DEM test was used to detect and classify oculomotor anomalies, which has good intra-subject test-retest reliability for all four of its scores when it is administered in an office setting, as well as good consistency in classifying patients as passes or fails [30,39]. Likewise, DEM outcomes have shown the capacity of identifying those children whose eye tracker recorded eye movement patterns showed slow reading rates [40]. However, the use of the DEM test in isolation for reaching a diagnosis of oculomotor anomaly has been suggested to be a potential source of error [41]. For this reason, in the current series, the DEM test was combined with the use of the NSUCO test, which has also been proven useful for detecting oculomotor alterations [42]. NSUCO and DEM tests can easily be applied in the clinical practice without the requirement of acquiring expensive technology.

A sample of children with completely healthy and emmetropic eyes was compared with two samples of groups, one including healthy children showing oculomotor anomalies according to DEM and NSUCO tests, and another one including children with specific learning disorders, including dyslexia, ADHD or dyspraxia. The evaluation with the NSUCO test showed significantly lower scorings for the ability, precision and head/body movement control associated to smooth pursuits and saccades in the OAG and LDG compared to the CG. Likewise, no significant differences were found when comparing the NSUCO outcomes obtained in the OAG and LDG. This confirms that the same levels of oculomotor alterations according to the NSUCO tests can be present in children with and without specific learning disorders. Similarly, DEM outcomes obtained in the CG differed significantly from those obtained in the OAG and LDG. As in previous studies [43], significantly longer times for reading sheet C was observed in the group with specific learning disorders. Furthermore, higher DEM pattern values and ratios, as well as a lower percentile for horizontal time, were found in the LDG compared to the CG. As happened with the NSUCO test outcomes, no significant differences were found between the OAG and LDG in any of the DEM parameters. Therefore, the same altered oculomotor, reading and visual processing behavior can be present in children with and without specific learning disorders. This suggests that alterations in oculomotricity are not specific of learning disorders and this aspect must not be considered as a unique criterion for diagnosing these conditions that require a multidisciplinary and complex diagnostic battery.

One potential limitation of the current comparative study could be the use of the DEM test, because despite its reliability in terms of consistency, some authors have stated that DEM test performances are not exactly correlated with saccadic eye movement skills, being more related to reading performance and visual processing speed [44]. This makes sense, considering that a reduction was also present in stereopsis (binocular vision processing) in the OAG and LDG. However, saccades are crucial in the reading process [7] and therefore are involved in part in the performance observed with the DEM test. For this reason, some authors have not recommended the use of only DEM tests to evaluate oculomotricity, with the recommendation of combining with other tests [43], such in our series. In the current series, the NSUCO test in which reading is not involved has confirmed the outcomes obtained with the DEM test. Therefore, oculomotor alterations were present as well as reading and visual processing deficits. This suggests that oculomotricity deficits can complicate reading and visual processing associated to the reading process as measured by the DEM test, but this should be investigated further in studies with specific design to test this hypothesis.

Another potential limitation of the current study is the use of clinical tests without eye tracking or videoculographic recording system, as this technology was not available in our clinical setting when the study was conducted. Future studies should be conducted to confirm the outcomes of the current study with this advanced eye tracking technologies. However, consistent diagnostic criteria for normality should be defined for the eye tracking outcomes, as the presence of significant differences between some groups compared to healthy control individuals only confirmed the presence of a difference but not the presence of an abnormality [16].

All oculomotor tests were performed in the current study by the same experienced examiner to avoid potential inter-observer variability which is normally present when performing clinical tests. This also may be considered a limitation of the study, as only one observer obtained the results reported in this study and it would be unknown if this may be extrapolated to other observers. However, the reliability of DEM and NSUCO tests for their use in the clinical practice, even in terms of inter-observer repeatability, have been demonstrated by several authors [31,41,43]. Finally, the reduced sample size in the current comparative study is another limitation that should considered, and therefore the conclusions of this study should be considered with care and confirmed in future studies with larger sample sizes.

## 5. Conclusions

Oculomotor anomalies can be present in children with and without specific learning disorders, and therefore cannot be used as diagnostic criteria of these complex types of disorders. This is contradictory to previous findings reported by different authors and should be confirmed in future trials with larger samples. More consistent diagnostic criteria are needed to characterize oculomotricity alterations and to define and predict the real impact of such alterations in reading performance. This would enable the defining of accurate standardized protocols for children with reading difficulties, involving different professionals in the process.

## Figures and Tables

**Table 1:** Summary of the main characteristics of the three groups evaluated in the study: CG, control group; OAG, group of children with oculomotor abnormalities; LDG, group of children with learning disorders.

Mean (SD) Median (Range)	CG (15)	OAG (18)	LDG (26)	*p*-Value
Age (years)	8.8 (1.6) 9.0 (6.0 to 11.0)	7.9 (1.3) 8.0 (6.0 to 11.0)	8.7 (2.1) 9.0 (6.0 to 13.0)	0.271
Sphere RE (D)	0.00 (0.00) 0.00 (0.00 to 0.00)	0.05 (0.24) 0.00 (0.00 to 1.00)	0.20 (0.89) 0.00 (−1.75 to 4.00)	0.191
Cylinder RE (D)	0.00 (0.00) 0.00 (0.00 to 0.00)	−0.03 (0.11) 0.00 (−0.50 to 0.00)	−0.32 (0.94) 0.00 (−4.00 to 0.00)	0.201
Sphere LE (D)	0.00 (0.00) 0.00 (0.00 to 0.00)	0.04 (0.18) 0.00 (0.00 to 0.75)	0.04 (1.20) 0.00 (−4.00 to 4.00)	0.571
Cylinder LE (D)	0.00 (0.00) 0.00 (0.00 to 0.00)	−0.01 (0.06) 0.00 (−0.25 to 0.00)	−0.21 (0.67) 0.00 (−3.00 to 0.00)	0.346
LogMAR CDVA RE	0.00 (0.01) 0.00 (0.00 to 0.05)	−0.04 (0.07) 0.00 (−0.20 to 0.05)	−0.02 (0.18) 0.00 (−0.05 to 0.30)	0.067
LogMAR CDVA LE	0.00 (0.03) 0.00 (0.00 to 0.10)	−0.04 (0.07) 0.00 (-0.20 to 0.05)	0.00 (0.06) 0.00 (-0.08 to 0.30)	0.050
Binocular LogMAR CDVA	0.0 (0.00) 0.00 (0.00 to 0.00)	−0.04 (0.07) 0.00 (−0.20 to 0.00)	−0.02 (0.05) 0.00 (−0.20 to 0.00)	0.057
Near cover test (Δ)	0.00 (0.00) 0.00 (0.00 to 0.00)	−1.67 (4.40) 0.00 (−8.00 to 10.00)	−2.73 (4.05) 0.00 (−10.00 to 4.00)	0.047 CG-OAG 0.534 CG-LDG 0.043 OAG-LDG 0.508
NPC break (cm)	4.27 (3.58) 5.00 (0.00 to 10.00)	4.17 (5.37) 0.00 (0.00 to 15.00)	6.58 (4.99) 5.50 (0.00 to 20.00)	0.200
NPC recovery (cm)	6.07 (4.80) 8.00 (0.00 to 14.00)	5.00 (6.15) 0.00 (0.00 to 16.00)	7.38 (6.56) 7.00 (3.00 to 25.00)	0.593
Stereopsis (sec arc)	20.0 (0.0) 20.0 (20.0 to 20.0)	31.9 (1.9) 32.0 (20.0 to 100.0)	64.4 (7.3) 40.0 (20.0 to 400.0)	<0.001 CG-OAG 0.018 CG-LDG <0.001 OAG-LDG 0.003

Abbreviations: RE, right eye; LE, left eye; D, diopter; CDVA, corrected distance visual acuity; NPC, near point of convergence. The results of the cover test were expressed as negative in the presence of exophoria and positive in the presence of esophoria.

**Table 2:** Summary of the results of the oculomotor examination with the NSUCO test in the three groups of children evaluated: CG, control group; OAG, group of children with oculomotor abnormalities; LDG, group of children with learning disorders.

Mean (SD) Median (Range)	CG (15)	OAG (18)	LDG (26)	*p*-Value
Smooth pursuits				
Ability	4.3 (1.1) 5.0 (2.0 to 5.0)	2.2 (1.3) 2.0 (1.0 to 5.0)	2.3 (1.3) 2.0 (1.0 to 5.0)	<0.001 CG-OAG <0.001 CG-LDG <0.001 OAG-LDG 0.999
Precision	4.1 (1.1) 4.0 (2.0 to 5.0)	2.3 (1.2) 2.0 (1.0 to 5.0)	2.3 (1.3) 2.0 (1.0 to 5.0)	<0.001 CG-OAG <0.001 CG-LDG <0.001 OAG-LDG 0.999
Movement head/body	4.2 (1.2) 5.0 (2.0 to 5.0)	2.1 (1.2) 2.0 (1.0 to 5.0)	1.5 (0.6) 1.0 (1.0 to 3.0)	<0.001 CG-OAG <0.001 CG-LDG <0.001 OAG-LDG 0.141
Saccades				
Ability	4.3 (1.0) 5.0 (2.0 to 5.0)	2.3 (1.2) 2.0 (1.0 to 5.0)	2.2 (0.9) 2.0 (1.0 to 4.0)	<0.001 CG-OAG <0.001 CG-LDG <0.001 OAG-LDG 0.999
Precision	4.3 (1.0) 5.0 (2.0 to 5.0)	2.2 (1.2) 2.0 (1.0 to 5.0)	2.4 (1.1) 2.0 (1.0 to 5.0)	<0.001 CG-OAG <0.001 CG-LDG <0.001 OAG-LDG 0.999
Movement head/body	4.2 (1.2) 5.0 (2.0 to 5.0)	2.1 (1.2) 2.0 (1.0 to 5.0)	1.6 (0.6) 1.5 (1.0 to 3.0)	<0.001 CG-OAG <0.001 CG-LDG <0.001 OAG-LDG 0.609

Abbreviations: SD, standard deviation.

**Table 3:** Summary of the results of the oculomotor examination with the Developmental Eye Movement (DEM) test in the three groups of children evaluated: CG, control group; OAG, group of children with oculomotor abnormalities; LDG, group of children with learning disorders.

Mean (SD) Median (Range)	CG (15)	OAG (18)	LDG (26)	*p*-Value
Time sheet A (s)	21.9 (6.5) 19.0 (16.0 to 38.0)	25.1 (7.1) 24.0 (17.0 to 43.0)	26.8 (8.6) 25.0 (11.3 to 42.8)	0.092
Time sheet B (s)	22.1 (6.1) 22.0 (15.0 to 37.0)	26.7 (7.1) 25.5 (19.0 to 47.0)	28.0 (8.6) 26.0 (11.4 to 44.0)	0.024 CG-OAG 0.075 CG-LDG 0.049 OAG-LDG 0.999
Time sheet C (s)	57.5 (23.1) 47.0 (38.4 to 107.0)	106.6 (50.2) 78.5 (46.0 to 240.0)	117.0 (62.8) 99.5 (30.3 to 247.0)	<0.001 CG-OAG <0.001 CG-LDG <0.001 OAG-LDG 0.999
DEM ratio	1.3 (0.3) 1.3 (1.0 to 2.0)	2.1 (1.0) 1.6 (1.0 to 4.7)	2.2 (1.1) 1.7 (1.0 to 5.1)	<0.001 CG-OAG 0.003 CG-LDG <0.001 OAG-LDG 0.999
Number of errors	4.7 (5.9) 4.0 (0.0 to 20.0)	5.4 (6.1) 2.5 (0.0 to 20.0)	11.2 (13.3) 5.0 (0.0 to 45.0)	0.631
Type of DEM pattern	1.0 (0.0) 1.0 (1.0 to 1.0)	2.4 (0.8) 2.0 (2.0 to 4.0)	2.5 (1.1) 2.0 (1.0 to 4.0)	<0.001 CG-OAG <0.001 CG-LDG <0.001 OAG-LDG 0.999
DEM percentile vertical times	52.3 (19.7) 52.0 (12.0 to 85.0)	44.6 (33.0) 35.0 (1.0 to 98.0)	33.6 (30.8) 23.5 (1.0 to 99.0)	0.385
DEM percentile horizontal time	51.7 (27.6) 55.0 (2.0 to 93.0)	14.7 (19.9) 10.5 (1.0 to 89.0)	17.0 (26.1) 2.0 (1.0 to 90.0)	<0.001 CG-OAG <0.001 CG-LDG <0.001 OAG-LDG 0.540

Abbreviations: SD, standard deviation.
